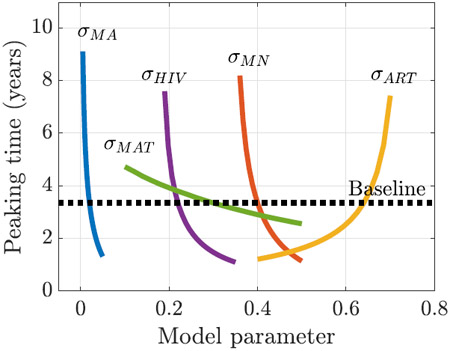# Staged progression epidemic models for the transmission of invasive nontyphoidal *Salmonella* (iNTS) with treatment

**DOI:** 10.3934/mbe.2021079

**Published:** 2021-01-28

**Authors:** Zhuolin Qu, Benjamin H. McMahon, Douglas J. Perkins, James M. Hyman

**Affiliations:** 1 Department of Mathematics, University of Texas at San Antonio, San Antonio 78202, TX, USA; 2 Theoretical Biology and Biophysics, Los Alamos National Laboratory, Los Alamos, NM, USA; 3 University of New Mexico, Center for Global Health, Department of Internal Medicine, NM, USA; 4 University of New Mexico-Kenya Global Health Programs, Kisumu and Siaya, Kenya; 5 Department of Mathematics, Tulane University, New Orleans 70112, LA, USA

**Keywords:** mathematical modeling, invasive nontyphoidal *Salmonella*, basic reproduction number, risk factors, asymptomatic carrier

## Abstract

We develop and analyze a stage-progression compartmental model to study the emerging invasive nontyphoidal *Salmonella* (iNTS) epidemic in sub-Saharan Africa. iNTS bloodstream infections are often fatal, and the diverse and non-specific clinical features of iNTS make it difficult to diagnose. We focus our study on identifying approaches that can reduce the incidence of new infections. In sub-Saharan Africa, transmission and mortality are correlated with the ongoing HIV epidemic and severe malnutrition. We use our model to quantify the impact that increasing antiretroviral therapy (ART) for HIV infected adults and reducing malnutrition in children would have on mortality from iNTS in the population. We consider immunocompromised subpopulations in the region with major risk factors for mortality, such as malaria and malnutrition among children and HIV infection and ART coverage in both children and adults. We parameterize the progression rates between infection stages using the branching probabilities and estimated time spent at each stage. We interpret the basic reproduction number ${ℛ}_{0}$ as the total contribution from an infinite infection loop produced by the asymptomatic carriers in the infection chain. The results indicate that the asymptomatic HIV+ adults without ART serve as the driving force of infection for the iNTS epidemic. We conclude that the worst disease outcome is among the pediatric population, which has the highest infection rates and death counts. Our sensitivity analysis indicates that the most effective strategies to reduce iNTS mortality in the studied population are to improve the ART coverage among high-risk HIV+ adults and reduce malnutrition among children.

## 1. Introduction

Typhoidal *Salmonella* and nontyphoidal *Salmonella* (NTS) are intracellular bacteria that cause significant global morbidity and mortality [1]. In industrialized countries, NTS is normally associated with zoonotic reservoirs and is transferred between humans and other animals through the fecal-oral route. Most infected individuals experience mild gastrointestinal illness, such as nausea, vomiting, profuse watery diarrhea, and abdominal pain. The infections are usually self-limiting, and antimicrobial treatment is not recommended for uncomplicated illness.

In Sub-Saharan Africa, in contrast to the rest of the world, a strain of NTS (ST-313) appears to be human-adapted, becoming a predominant cause of invasive nontyphoidal *Salmonella* (iNTS) bloodstream infections [2,3]. The clinical features of iNTS disease are diverse and non-specific, and diarrhea is often absent. The symptoms range from hepatosplenomegaly to respiratory symptoms. These symptoms overlap with pneumonia and malaria clinical presentations in Sub-Saharan Africa, and iNTS is often misdiagnosed. Empirical diagnosis often fails to identify and treat iNTS disease [4]. Even when the microbiologically confirmed cases are treated with appropriate antimicrobial drugs, there is a high case fatality of 22–47% [2, 5, 6].

The emergence of iNTS ST-313 in sub-Saharan Africa is thought to be due to the large proportion of the African population with some degree of immune suppression or impairment caused by malnutrition or comorbidity with untreated HIV and other infections [7,8]. The risk factors in children include HIV infection, malaria, and malnutrition, and the main risk factor in adults is the advanced HIV infection. Among the HIV-infected adults, before the antiretroviral therapy era, 20–40% of survivors had recurrence; despite the treatment, up to 25% of patients had several recurrences [5, 9].

There is some evidence showing that iNTS may have evolved to transmit from person to person [10-12]. Moreover, asymptomatic carriers can shed NTS into the environment and infect other children in nearby homes. These asymptomatic carriers create a reservoir of iNTS infections for community-acquired iNTS bacteremia in children [13, 14].

Multidrug-resistant iNTS is a challenge for the local healthcare system and can be a major factor in the high prevalence [12]. *Salmonella* was once susceptible to a broad range of affordable and effective antimicrobial drugs. Recently, multidrug-resistant strains have emerged, and a large proportion of the infection are resistant to three or more commonly available antibiotics.

Mathematical models are tools to help understand the dynamics of epidemics and guide the mitigation efforts [15, 16]. Both compartmental differential equation models and stochastic individual-based models have been developed to clarify the zoonotic *Salmonella* transmission dynamics. These models can inform public health workers and help eradicate the infection from a population. For example, in [17], a compartmental model simulates a *Salmonella* Typhimurium infection in swine in Great Britain. In this model, the infected pigs are grouped into susceptible, latent, infectious (shedder), and carrier stage compartments. In [18, 19], Susceptible-Infectious-Recovered (SIRS) type compartmental models were used to simulate the transmission dynamics of *Salmonella* infection in dairy herds. In [20], a multidisciplinary approach involves quantitative PCR, and probabilistic models are proposed to study the spatial and stochastic nature of within-in host dynamics for *Salmonella* Typhimurium infection.

Although there are many *Salmonella* models in animals, there are very few models for iNTS within human populations in the developing world, such as Sub-Saharan Africa. In [21], a Susceptible-Infectious-Recovered type differential equation model is proposed as the first model for iNTS dynamics in this high-risk population, with the focus on the cost-benefit analysis for different scenarios of diagnostic deployment and their impact on antimicrobial treatment for the patients.

We propose a staged progression ODE model to help understand the transmission dynamics of iNTS and inform effective public health control policy. We formulate the model for an immunocompromised population in Sub-Saharan Africa and account for different risk factors, including untreated HIV/AIDS infections. The model accounts for multiple infection stages, including asymptomatic carriers.

After describing the proposed model (Section 2), we derive the disease basic reproduction number and analyze how much each infection stage contributes to the spread of the iNTS (Section 3). We then simulate a baseline scenario in Siaya County, Kenya (Section 4.1), characterize the impact of the different risk factors towards the local epidemic through sensitivity analysis, and inform potential mitigation strategies (Section 4.2).

## 2. Staged progression model with treatment

**Figure 1.**
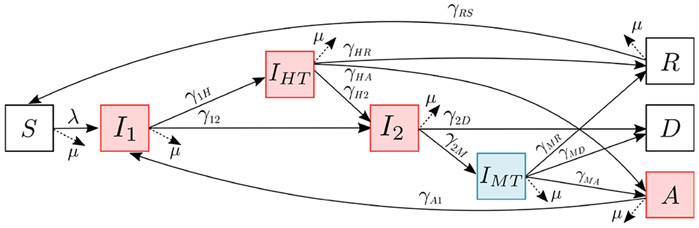
Disease progression model for iNTS with treatments. The red boxes ($I_{1}$, $I_{HT}$, $I_{2}$ A) are infectious stages, and the blue box ($I_{MT}$) is infected but not infectious. The progression rate from compartment j to compartment k is given by $\gamma_{jk}$.

Our staged progression compartmental model (Figure 1) accounts for two infection stages and two treatment stages with different outcome statuses. The susceptible population, S, is infected at a rate of infection λ and progresses to an early-stage infection, $I_{1}$, with mild symptoms. Some of the infected individuals receive home treatment, entering stage $I_{HT}$ (at rate $\gamma_{1H}$). The outcome of home treatment include recovered R (at rate $\gamma_{HR}$), asymptomatic A (at rate $\gamma_{HA}$), or severe infection status $I_{2}$ (at rate $\gamma_{H2}$) with acute/serious symptoms. The infected people not receiving home treatment enter severe infection $I_{2}$ directly at rate $\gamma_{12}$.

Some of the severely sick population $I_{2}$ are treated in a medical facility and enter the compartment $I_{MT}$ at the rate $\gamma_{2M}$. They then either recover and enter R at the rate $\gamma_{MR}$, die and enter D at rate $\gamma_{MD}$, or become an asymptomatic carrier A at rate $\gamma_{MA}$. We assume that those with severe symptoms who do not receive medical treatment will suffer mortality at the rate $\gamma_{2D}$. The recovered population, R, obtains some temporary immunity from the infection and becomes susceptible S again at the rate $\gamma_{RS}$.

Home treatment $I_{HT}$ is common in Sub-Saharan Africa, and antibiotics are easily available over the counter without a prescription for self-treatment. Inappropriate use of these drugs may contribute to the emergence of multidrug-resistant phenotypes of iNTS [13]. Also, less expensive generic drugs of variable quality for treating bacterial infections could also be contributing to the increasing resistance. Although we do not explicitly model the emergence of resistant strains in the current model, we will be considering these factors in future versions of the model.

In Sub-Saharan Africa, iNTS infection rates peak first in young children ⩽ 5 years old and then again in adults 25–40 years old [22]. The first peak is related to malaria, malnutrition, and HIV for young children, while the second peak is due to advanced HIV infection in the adults.

We account for the immunocompromised adults with advanced HIV infection who become asymptomatic carriers A. These asymptomatic carriers feel well but are still transmitting the pathogen. They are often receiving effective home treatment in early-stage infection $I_{HT}$ or medical treatment in severe infection $I_{MT}$, and they still retain a relatively low pathogen load after the treatment. If these infected carriers are on Antiretroviral Therapy (ART) HIV treatment, then their immune system is usually strong enough to fight off the iNTS bacteria. However, if they are not on ART, then eventually the bacterial load increases until it breaks down their immune system, and they become sick again (at rate $\gamma_{A1}$). Although these asymptomatic carriers feel well, they are still infectious and constantly shedding the disease into the environment without being detected. Thus, these asymptomatic carriers act as a reservoir for the iNTS emergence and transmission [8].

Because the iNTS prevalence is highest in age groups ⩽ 5 and 25–40 years old, we explicitly account for these age groups in the model. The older age group also can capture the impact of asymptomatic HIV-infected adults. We divide the population into the following groups, denoted by upper index κ: HIV− adults (κ=1), HIV+ with ART adults (κ=2), HIV+ not treated adults (κ=3), and young children ⩽ 5 years old (κ=4). For each group κ, we model the disease dynamics using the same framework shown in Figure 1 but with different progression rates between the compartments. For simplicity of notation, we omit the group index κ when the formulas are the same for all the groups unless there could be some ambiguity in the notation. A description of all the state variables is in Table 1.

### 2.1. Progression rates and branching process

**Figure 2.**
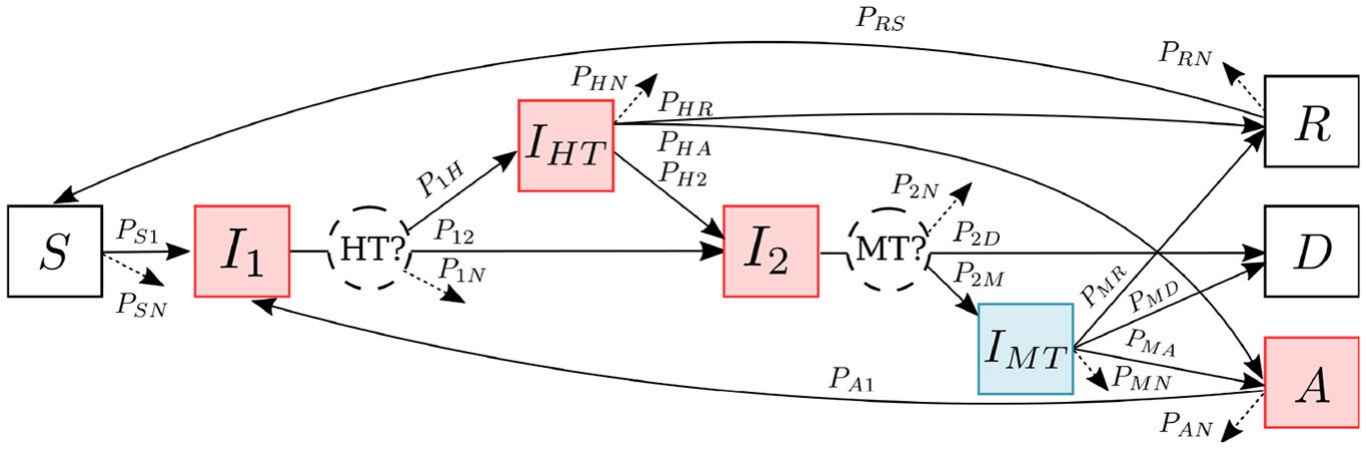
Branching process for disease progression model for iNTS. The red boxes ($I_{1}$, $I_{HT}$, $I_{2}$, A) are the infectious stages, and the blue box ($I_{MT}$) is infected but not infectious. Branching probability $P_{jk}$ is the fraction of people who progress from state j to state k. The progression rates $\gamma_{jk}$ in Figure 1 are defined by Eq (2.2) using the branching fractions $P_{jk}$ and the time spent in each compartment.

The rates that people advance between the compartments in Figure 1 depend on the average time people spend in each compartment and the branching probabilities which are distributed across all possible downstream compartments (Figure 2).

For each group κ, we first define the branching probabilities, $P_{ij}^{\kappa }$, as the fraction of people who progress from state i to state j. These exit probabilities sum to unity (Eq (2.1)) and, even though this constraint could reduce the number of variables, we keep all of them in the equations to simplify the notation. Then, we define $\tau_{i}^{\kappa }$ as the average time spent in a state i. Assuming an exponential distribution for the time spent in a stage i, we can derive the progression rates $\gamma_{ij}^{\kappa }$ in terms of the ratios of the branching probabilities and time spent in the upstream stage $\tau_{i}^{\kappa }$, as shown in Eq (2.2).

We find that this approach is more intuitive and less prone to errors than defining the progression rates directly, especially when having multiple pathways from one compartment to another.


(2.1) $$P_{S1}+P_{SN}=1,\;P_{12}+P_{1H}+P_{1N}=1,\;P_{2D}+P_{2M}+P_{2N}=1,\;P_{A1}+P_{AN}=1,\;P_{MR}+P_{MD}+P_{MA}+P_{MN}=1,\;P_{HR}+P_{HA}+P_{H2}+P_{HN}=1,\;P_{RS}+P_{RN}=1.$$

(2.2) $$\gamma_{1H}=P_{1H}∕\tau_{1},\;\gamma_{HR}=P_{HR}∕\tau_{H},\;\gamma_{MR}=P_{MR}∕\tau_{M},\;\;\gamma_{12}=P_{12}∕\tau_{1},\;\gamma_{HA}=P_{HA}∕\tau_{H},\;\gamma_{MD}=P_{MD}∕\tau_{M},\;\gamma_{2D}=P_{2D}∕\tau_{2},\;\gamma_{H2}=P_{H2}∕\tau_{H},\;\gamma_{MA}=P_{MA}∕\tau_{M},\;\gamma_{2M}=P_{2M}∕\tau_{2},\;\gamma_{RS}=P_{RS}∕\tau_{R},\;\gamma_{A1}=P_{A1}∕\tau_{A},$$

(2.3) $$\mu =P_{1N}∕\tau_{1}=P_{HN}∕\tau_{H}=P_{2N}∕\tau_{2}=P_{MN}∕\tau_{M}=P_{AN}∕\tau_{A}=P_{RN}∕\tau_{R}$$

### 2.2. Force of infection

We model the force of infection rates for all the susceptible groups, $\lambda^{\kappa }$, as the summation of the sources of infection from each infectious stage among all the groups:

(2.4) $$\lambda^{\kappa }=c^{\kappa }\beta^{\kappa }\frac{\sum_{\ell =1}^{4}c^{\ell }(I_{1}^{\ell }+I_{HT}^{\ell }+I_{2}^{\ell }+A^{\ell })}{\sum_{\ell =1}^{4}c^{\ell }(S^{\ell }+I_{1}^{\ell }+I_{HT}^{\ell }+I_{2}^{\ell }+A^{\ell }+R^{\ell })},\;\kappa =1,\ldots 4,$$

where $c^{\kappa }$ is the number of contacts that an individual in group κ has per day. We assume a homogenously mixing population and that the proportion of the contacts with an infectious person is approximated by the last term in the Eq (2.4). This proportion is defined by dividing the total number of contacts from the infectious population per day (the numerator) by the total number of contacts from all the active population per day (the denominator). We have assumed that the infectious people do not change their behavior until being admitted to the medical facility (stage $I_{MT}$). Once in a medical facility, then we assume they no longer have contacts with the general population and are not part of force of infection.

For simplicity and the lack of data, in our study, we assume that contacts in different groups are equal, $c=c^{\kappa }$ for κ=1,…4. Under this assumption, the force of infection in Eq (2.4) can be reduced to

$$\lambda^{\kappa }=c\;\beta^{\kappa }\frac{\sum_{\ell =1}^{4}I_{1}^{\ell }+I_{HT}^{\ell }+I_{2}^{\ell }+A^{\ell }}{\sum_{\ell =1}^{4}S^{\ell }+I_{1}^{\ell }+I_{HT}^{\ell }+I_{2}^{\ell }+A^{\ell }+R^{\ell }},\;\kappa =1,\ldots 4.$$

We assume that the transmissibility per contact $\beta^{\kappa }$ for each subpopulation is approximately constant through different infection stages. For different groups κ=1,⋯,4, the transmissibility $\beta^{\kappa }$ varies. This difference reflects the average level of immune-competency of susceptible adults or children in the corresponding group.

Following the analysis in Feasey et al. [23], within the young child group (κ=4), since the risk factors, such as malaria and HIV, are at endemic state in the studying region, we formulate the averaged susceptibilities as the contribution of prevalences of these factors,

$$\beta^{4}=\beta_{0}^{4}(0.56\;\sigma_{MA}+0.25\;\sigma_{HIV}^{C}+0.2\;\sigma_{MN}),$$

where $\sigma_{MA}$ and $\sigma_{MN}$ are the prevalence of malaria and malnutrition, respectively, in children under age five. The coefficient $\sigma_{HIV}^{C}$ is the HIV prevalence in the child group, which depends on the fraction of non-ART-treated HIV adults ($1-\sigma_{ART}$) and the maternal transmission rate ($\sigma_{MAT}$),

$$\sigma_{HIV}^{C}=\sigma_{HIV}^{A}(1-\sigma_{ART})\sigma_{MAT}.$$

This approach results in an estimated prevalence of 2.4% in children in the baseline scenario. There is very little correlation between iNTS incidence and ART treatment in children [7,24], and it is neglected in the model.

The resulting model for iNTS transmission among high-risk cohorts in Sub-Saharan Africa is expressed as a system of differential equations for each population group κ,

(2.5) $${\dot{S}}^{\kappa }=\mu^{\kappa }(S_{0}^{\kappa }-S^{\kappa })-\lambda^{\kappa }S^{\kappa }+\gamma_{RS}^{\kappa }R^{\kappa },\;\;{\dot{I}}_{1}^{\kappa }=\lambda^{\kappa }S^{\kappa }+\gamma_{A1}^{\kappa }A^{\kappa }-(\gamma_{1H}^{\kappa }+\gamma_{12}^{\kappa })I_{1}^{\kappa }-\mu^{\kappa }I_{1}^{\kappa },\;{\dot{I}}_{HT}^{\kappa }=\gamma_{1H}^{\kappa }I_{1}^{\kappa }-(\gamma_{HR}^{\kappa }+\gamma_{HA}^{\kappa }+\gamma_{H2}^{\kappa })I_{HT}^{\kappa }-\mu^{\kappa }I_{HT}^{\kappa },\;\;{\dot{I}}_{2}^{\kappa }=\gamma_{H2}^{\kappa }I_{HT}^{\kappa }+\gamma_{12}^{\kappa }I_{1}^{\kappa }-(\gamma_{2D}^{\kappa }+\gamma_{2M}^{\kappa })I_{2}^{\kappa }-\mu^{\kappa }I_{2}^{\kappa },\;\;\;\;\;\kappa =1,\ldots 4.\;{\dot{I}}_{MT}^{\kappa }=\gamma_{2M}^{\kappa }I_{2}^{\kappa }-(\gamma_{MR}^{\kappa }+\gamma_{MD}^{\kappa }+\gamma_{MA}^{\kappa })I_{MT}^{\kappa }-\mu^{\kappa }I_{MT}^{\kappa },\;\;{\dot{A}}^{\kappa }=\gamma_{HA}^{\kappa }I_{HT}^{\kappa }+\gamma_{MA}^{\kappa }I_{MT}^{\kappa }-\gamma_{A1}^{\kappa }A^{\kappa }-\mu^{\kappa }A^{\kappa },\;\;{\dot{R}}^{\kappa }=\gamma_{HR}^{\kappa }I_{HT}^{\kappa }+\gamma_{MR}^{\kappa }I_{MT}^{\kappa }-\gamma_{RS}^{\kappa }R^{\kappa }-\mu^{\kappa }R^{\kappa },\;\;{\dot{D}}^{\kappa }=\gamma_{2D}^{\kappa }I_{2}^{\kappa }+\gamma_{MD}^{\kappa }I_{MT}^{\kappa },$$

all the progression rates are defined in Eqs (2.2) and (2.3), and all the model parameters are summarized in Table 2.

When there is no iNTS epidemic present in the population, the susceptible population $S^{\kappa }$ is balanced at a steady state level $S_{0}^{\kappa }$ through a constant birth and natural removing rate $\mu^{\kappa }$. We consider the migration (removal) rate $\mu^{\kappa }$ in the equations to account for non-iNTS mortality and the natural aging out of the age cohort.

## 3. Basic reproduction number and steady states

We use the next generation matrix approach to define the basic reproduction number ${ℛ}_{0}$ for the system Eq (2.5). The resulting formula can be expressed as a weighted sum of the subgroup basic reproduction numbers, ${ℛ}_{0}^{\kappa }$, κ=1,…,4. The subgroup basic reproduction numbers, ${ℛ}_{0}^{\kappa }$, must account for recurring infections, and we are able to explain the formula in terms of the sum of terms in an infinite recurrence relationship.

### 3.1. Calculations of basic reproduction number ${ℛ}_{0}$

Following the next generation matrix approach, we consider the equations in Eq (2.5) that are associated with the infected groups: $X={\left(X^{1},X^{2},X^{3},X^{4}\right)}^{T}$, where $X^{\kappa }=(I_{1}^{\kappa },I_{HT}^{\kappa },I_{2}^{\kappa },I_{MT}^{\kappa },A^{\kappa })$. Next, the right-hand sides of equations for the infected groups are split into the infection part, ${ℱ}^{\kappa }$, and transition part ${𝒱}^{\kappa }$:

$$\frac{d}{dt}\left(\begin{array}{c} I_{1}^{\kappa }\\ I_{HT}^{\kappa }\\ I_{2}^{\kappa }\\ I_{MT}^{\kappa }\\ A^{\kappa } \end{array}\right)=\left(\begin{array}{c} c\beta^{\kappa }\;\frac{\sum_{\ell =1}^{4}I_{1}^{\ell }+I_{HT}^{\ell }+I_{2}^{\ell }+A^{\ell }}{\sum_{\ell =1}^{4}S^{\ell }+I_{1}^{\ell }+I_{HT}^{\ell }+I_{2}^{\ell }+A^{\ell }+R^{\ell }}S^{\kappa }\\ 0\\ 0\\ 0\\ 0 \end{array}\right)-\left(\begin{array}{c} (\gamma_{1H}^{\kappa }+\gamma_{12}^{\kappa })I_{1}^{\kappa }-\gamma_{A1}^{\kappa }A^{\kappa }+\mu^{\kappa }I_{1}^{\kappa }\\ (\gamma_{HR}^{\kappa }+\gamma_{HA}^{\kappa }+\gamma_{H2}^{\kappa })I_{HT}^{\kappa }-\gamma_{1H}^{\kappa }I_{1}^{\kappa }+\mu^{\kappa }I_{HT}^{\kappa }\\ (\gamma_{2D}^{\kappa }+\gamma_{2M}^{\kappa })I_{2}^{\kappa }-\gamma_{H2}^{\kappa }I_{HT}^{\kappa }-\gamma_{12}^{\kappa }I_{1}^{\kappa }+\mu^{\kappa }I_{2}^{\kappa }\\ (\gamma_{MR}^{\kappa }+\gamma_{MD}^{\kappa }+\gamma_{MA}^{\kappa })I_{MT}^{\kappa }-\gamma_{2M}^{\kappa }I_{2}^{\kappa }+\mu^{\kappa }I_{MT}^{\kappa }\\ \gamma_{A1}^{\kappa }A^{\kappa }-\gamma_{HA}^{\kappa }I_{HT}^{\kappa }-\gamma_{MA}^{\kappa }I_{MT}^{\kappa }+\mu^{\kappa }A^{\kappa } \end{array}\right)\;\;=:{ℱ}^{\kappa }-{𝒱}^{\kappa },\;\;\kappa =1,\ldots ,4.$$

These equations are then linearized at the disease-free equilibrium (DFE) $X_{0}^{\kappa }\;=(S_{0}^{\kappa },0,0,0,0,0,0)$, κ=1,2,3,4. That is, we define Jacobian matrices of $ℱ≔({ℱ}^{1};{ℱ}^{2};{ℱ}^{3};{ℱ}^{4})$ and $𝒱≔({𝒱}^{1};{𝒱}^{2};{𝒱}^{3};{𝒱}^{4})$ evaluate them at the DFE to obtain

$$J_{ℱ}≔\frac{\partial ℱ}{\partial X}\left(\begin{array}{cccc} D_{ℱ}^{1} & D_{ℱ}^{1} & D_{ℱ}^{1} & D_{ℱ}^{1}\\ D_{ℱ}^{2} & D_{ℱ}^{2} & D_{ℱ}^{2} & D_{ℱ}^{2}\\ D_{ℱ}^{3} & D_{ℱ}^{3} & D_{ℱ}^{3} & D_{ℱ}^{3}\\ D_{ℱ}^{4} & D_{ℱ}^{4} & D_{ℱ}^{4} & D_{ℱ}^{4} \end{array}\right)\;\;\text{and}\;\;J_{𝒱}≔\frac{\partial 𝒱}{\partial X}=\left(\begin{array}{cccc} {𝒟}_{𝒱}^{1} & \; & \; & \;\\ \; & D_{𝒱}^{2} & \; & \;\\ \; & \; & D_{𝒱}^{3} & \;\\ \; & \; & \; & D_{𝒱}^{4} \end{array}\right),$$

where

$$D_{ℱ}^{\kappa }=\left(\begin{array}{ccccc} \varphi^{\kappa } & \varphi^{\kappa } & \varphi^{\kappa } & 0 & \varphi^{\kappa }\\ 0 & 0 & 0 & 0 & 0\\ 0 & 0 & 0 & 0 & 0\\ 0 & 0 & 0 & 0 & 0\\ 0 & 0 & 0 & 0 & 0 \end{array}\right),\;\;\varphi^{\kappa }=c\beta^{\kappa }\frac{S_{0}^{\kappa }}{N_{0}},\;\;\kappa =1,\cdots ,4,\;\;N_{0}=\sum_{\kappa =1}^{4}S_{0}^{\kappa },$$

and

$$D_{𝒱}^{\kappa }=\left(\begin{array}{ccccc} \gamma_{12}^{\kappa }+\gamma_{1H}^{\kappa }+\mu^{\kappa } & 0 & 0 & 0 & -\gamma_{A1}^{\kappa }\\ -\gamma_{1H}^{\kappa } & \gamma_{H2}^{\kappa }+\gamma_{HA}^{\kappa }+\gamma_{HR}^{\kappa }+\mu^{\kappa } & 0 & 0 & 0\\ -\gamma_{12}^{\kappa } & -\gamma_{H2}^{\kappa } & \gamma_{2D}^{\kappa }+\gamma_{2M}^{\kappa }+\mu^{\kappa } & 0 & 0\\ 0 & 0 & -\gamma_{2M}^{\kappa } & \gamma_{MA}^{\kappa }+\gamma_{MD}^{\kappa }+\gamma_{MR}^{\kappa }+\mu^{\kappa } & 0\\ 0 & -\gamma_{HA}^{\kappa } & 0 & -\gamma_{MA}^{\kappa } & \gamma_{A1}^{\kappa }+\mu^{\kappa } \end{array}\right).$$

The basic reproduction number is calculated as the spectral radius of the next generation matrix $J_{ℱ}J_{𝒱}^{-1}$. Note that the matrix $J_{𝒱}$ is defined in terms of the transition rates $\gamma_{*}$, and the next generation matrix is defined in terms of its inverse, $J_{𝒱}^{-1}$. Thus, the resulting formulas for ${ℛ}_{0}$ are better understood in terms of $\tau_{*}$ and $P_{*}$ as defined in Eqs (2.1) to (2.3).

After long algebraic manipulations, we obtain

(3.1) $${ℛ}_{0}≔\text{Spectral Radius of}\;(J_{ℱ}J_{𝒱}^{-1})=\sum_{\kappa =1}^{4}\frac{S_{0}^{\kappa }}{N_{0}}{ℛ}_{0}^{\kappa },$$

where

(3.2) $${ℛ}_{0}^{\kappa }=c\beta^{\kappa }\frac{\tau_{1}^{\kappa }+P_{1H}^{\kappa }\tau_{H}^{\kappa }\left(P_{12}^{\kappa }+P_{1H}^{\kappa }P_{H2}^{\kappa }\right)\tau_{2}^{\kappa }+\left(P_{1H}^{\kappa }P_{HA}^{\kappa }+P_{1H}^{\kappa }P_{H2}^{\kappa }P_{2M}^{\kappa }P_{MA}^{\kappa }+P_{12}^{\kappa }P_{2M}^{\kappa }P_{MA}^{\kappa }\right)\tau_{A}^{\kappa }}{1-P_{A1}^{\kappa }\left(P_{1H}^{\kappa }P_{HA}^{\kappa }+P_{1H}^{\kappa }P_{H2}^{\kappa }P_{2M}^{\kappa }P_{MA}^{\kappa }+P_{12}^{\kappa }P_{2M}^{\kappa }P_{MA}^{\kappa }\right)}.$$

The obtained basic reproduction number ${ℛ}_{0}$ Eq (3.1) for the entire population is a weighted average of the contributions from each population group (${ℛ}_{0}^{\kappa }$) with the weights being the fractions of the population in the corresponding group. Since we partition the population into groups of subpopulation with different transmission parameters, our model structure is comparable to the differential infectivity model studied in [40], where the basic reproduction number has a similar pattern of weighted average.

### 3.2. Interpretation of basic reproduction numbers ${ℛ}_{0}^{\kappa }$ for subpopulation

We have written the basic reproduction number ${ℛ}_{0}^{\kappa }$ in terms of branching probabilities rather than progression rates, and this allows us to give more intuitive interpretations of the quantity. The factor $c\beta^{\kappa }$ in the reproduction number represents the number of contacts per day times the probability of infection per contact with someone in age-group κ. Together with the terms in the numerator of Eq (3.2), it gives the total contribution of infection from different infectious stages (detailed later).

The denominator in the formula Eq (3.2) for ${ℛ}_{0}^{\kappa }$, however, can look perplexing at first. This can be interpreted as a scaling factor to account for the possibility that the asymptomatic individuals can have recurrent infection ($A\to I_{1}$) and continue shedding to the population.

From Eq (3.2), we recognize that the coefficient factors for $\tau_{*}^{\kappa }$ represent the probabilities of transitions between the initial infected stage $I_{1}^{\kappa }$ and the corresponding infectious stage, that is

(3.3) $${ℛ}_{0}^{\kappa }=c\beta^{\kappa }\frac{\tau_{1}^{\kappa }+Prob\;(I_{1}^{\kappa }\to I_{HT}^{\kappa })\;\tau_{H}^{\kappa }+Prob\;(I_{1}^{\kappa }\to I_{2}^{\kappa })\;\tau_{2}^{\kappa }+Prob\;(I_{1}^{\kappa }\to A^{\kappa })\;\tau_{A}^{\kappa }}{1-Prob\;(I_{1}^{\kappa }\to A^{\kappa })\;P_{A1}^{\kappa }},$$

where

(3.4) Prob(I1κ→IHTκ)=P1Hκ,Prob(I1κ→I2κ)=P12κ+P1HκPH2κ,Prob(I1κ→Aκ)={Route#1I1κ→IHTκ→Aκ,P1HκPHAκ,Route#2I1κ→IHTκ→I2κ→IMTκ→Aκ,P1HκPH2κP2MκPMAκ,Route#3I1κ→I2κ→IMTκ→Aκ,P12κP2MκPMAκ,}=P1HκPHAκ+P1HκPH2κP2MκPMAκ+P12κP2MκPMAκ.

When being infectious, one first spends $\tau_{1}^{\kappa }$ amount of time in stage $I_{1}^{\kappa }$, then, with probability $Prob\;(I_{1}^{\kappa }\to I_{HT}^{\kappa })$, one progresses to $I_{HT}^{\kappa }$ stage and spend $\tau_{H}^{\kappa }$ amount of time there. Similarly, with probability $Prob\;(I_{1}^{\kappa }\to I_{2}^{\kappa })$ and $Prob\;(I_{1}^{\kappa }\to A^{\kappa })$, one progresses to stages $I_{2}^{\kappa }$ and $A^{\kappa }$ and spends $\tau_{2}^{\kappa }$ and $\tau_{A}^{\kappa }$ amount of time, respectively. For convenience, we define the expected duration of time one spends in a stage Δ as

(3.5) $${\bar{\tau }}_{\Delta }^{\kappa }=Prob\;(I_{1}^{\kappa }\to \Delta )\tau_{\Delta }^{\kappa },\;\;\Delta \in \{I_{1}^{\kappa },I_{HT}^{\kappa },I_{2}^{\kappa },I_{MT}^{\kappa },R^{\kappa },A^{\kappa }\},$$

where the probabilities $Prob\;(I_{1}^{\kappa }\to I_{HT}^{\kappa })$, $Prob\;(I_{1}^{\kappa }\to I_{2}^{\kappa })$, and $Prob\;(I_{1}^{\kappa }\to A^{\kappa })$ are defined in Eq (3.4), and

(3.6) $$Prob\;(I_{1}^{\kappa }\to I_{1}^{\kappa })=1,\;Prob\;(I_{1}^{\kappa }\to I_{MT}^{\kappa })=Prob\;(I_{1}^{\kappa }\to I_{2}^{\kappa })\;P_{2M}^{\kappa }=(P_{12}^{\kappa }+P_{1H}^{\kappa }P_{H2}^{\kappa })\;P_{2M}^{\kappa },\;Prob\;(I_{1}^{\kappa }\to R^{\kappa })=P_{1H}^{\kappa }P_{HR}^{\kappa }+P_{1H}^{\kappa }P_{H2}^{\kappa }P_{2M}^{\kappa }P_{MR}^{\kappa }+P_{12}^{\kappa }P_{2M}^{\kappa }P_{MR}^{\kappa }.$$

Then the average infectious time in one disease cycle, ${\bar{\tau }}_{I}^{\kappa }$, is

(3.7) $${\bar{\tau }}_{I}^{\kappa }≔{\bar{\tau }}_{1}^{\kappa }+{\bar{\tau }}_{H}^{\kappa }+{\bar{\tau }}_{2}^{\kappa }+{\bar{\tau }}_{A}^{\kappa }.$$

Thus, the average number of new secondary cases that an infectious individual could produce in one disease cycle is the summation of all the new cases in four infectious compartments

$${\left({ℛ}_{0}^{\kappa }\right)}^{\text{cycle}}≔c\beta^{\kappa }{\bar{\tau }}_{I}^{\kappa }.$$

Since the basic reproduction number accounts for all the secondary infections that happened during the entire infection duration, we need to include the recurrent infection when an asymptomatic person $A^{\kappa }$ becomes $I_{1}^{\kappa }$ again. Note that this is different from being reinfected after recovery ($R^{\kappa }\to I_{1}^{\kappa }$), which is considered as a separate infectious period. The probability of having a recurrent infection is

(3.8) $${𝒫}_{\gamma }^{\kappa }=Prob\;(I_{1}^{\text{cycle}\;1}\to A)\cdot Prob\;(A\to I_{1}^{\text{cycle}\;2})=Prob\;(I_{1}^{\kappa }\to A^{\kappa })\cdot P_{A1}^{\kappa }.$$

Then, with probability ${𝒫}_{\gamma }^{\kappa }$, the infected individual would have another infectious onset and create another ${\left({ℛ}_{0}^{\kappa }\right)}^{\text{cycle}}$ number of new infections in the second cycle. This process may repeat again and again with diminishing probability, and the ${ℛ}_{0}$ of the entire process is the summation of the resulting infinite geometric series,

$${ℛ}_{0}^{\kappa }=\underset{1\text{st cycle of infection}}{\underset{︸}{{\left({ℛ}_{0}^{\kappa }\right)}^{\text{cycle}}}}+\underset{2\text{nd cycle}}{\underset{︸}{{𝒫}_{\gamma }^{\kappa }{\left({ℛ}_{0}^{\kappa }\right)}^{\text{cycle}}}}+\cdots +\underset{n^{\text{th}}\;\text{cycle}}{\underset{︸}{{\left({𝒫}_{\gamma }^{\kappa }\right)}^{n-1}{\left({ℛ}_{0}^{\kappa }\right)}^{\text{cycle}}}}+\cdots =\frac{{\left({ℛ}_{0}^{\kappa }\right)}^{\text{cycle}}}{1-{𝒫}_{\gamma }^{\kappa }}=Eq\;(3.3).$$

This recovers the basic reproduction number obtained through the next generation matrix approach in Eq (3.3).

### 3.3. Endemic steady state

Setting the right-hand side of the system Eq (2.5) and substituting all the rates by probabilities as defined in Eqs (2.1) to (2.3), we obtain the endemic steady state for each subpopulation group:

(3.9) $${\left(I_{1}^{\kappa }\right)}_{EE}=\frac{\mu^{\kappa }\left(S_{0}^{\kappa }-S_{EE}^{\kappa }\right)}{1-{𝒫}_{\theta }^{\kappa }}{\bar{\tau }}_{1}^{\kappa },\;\;\;\;\;\;\;\;\;{\left(I_{HT}^{\kappa }\right)}_{EE}=\frac{\mu^{\kappa }\left(S_{0}^{\kappa }-S_{EE}^{\kappa }\right)}{1-{𝒫}_{\theta }^{\kappa }}{\bar{\tau }}_{H}^{\kappa },\;{\left(I_{2}^{\kappa }\right)}_{EE}=\frac{\mu^{\kappa }\left(S_{0}^{\kappa }-S_{EE}^{\kappa }\right)}{1-{𝒫}_{\theta }^{\kappa }}{\bar{\tau }}_{2}^{\kappa },\;\;\;\;\;\;\;\;\;{\left(I_{MT}^{\kappa }\right)}_{EE}=\frac{\mu^{\kappa }\left(S_{0}^{\kappa }-S_{EE}^{\kappa }\right)}{1-{𝒫}_{\theta }^{\kappa }}{\bar{\tau }}_{M}^{\kappa },\;{\left(A^{\kappa }\right)}_{EE}=\frac{\mu^{\kappa }\left(S_{0}^{\kappa }-S_{EE}^{\kappa }\right)}{1-{𝒫}_{\theta }^{\kappa }}{\bar{\tau }}_{A}^{\kappa },\;\;\;\;\;\;\;\;\;{\left(R^{\kappa }\right)}_{EE}=\frac{\mu^{\kappa }\left(S_{0}^{\kappa }-S_{EE}^{\kappa }\right)}{1-{𝒫}_{\theta }^{\kappa }}{\bar{\tau }}_{R}^{\kappa },$$

where ${\bar{\tau }}_{\Delta }^{\kappa }$ are defined in Eq (3.5) and

$${𝒫}_{\theta }^{\kappa }=Prob\;(I_{1}^{\kappa }\to A^{\kappa })P_{A1}^{\kappa }+Prob\;(I_{1}^{\kappa }\to R^{\kappa })P_{RS}^{\kappa }$$

is the probability of an infected individual to survive the infection and becomes either asymptomatic (first term) or recovered (second term), and the susceptible subpopulation at endemic state, ($S_{EE}^{1}$, $S_{EE}^{2}$, $S_{EE}^{3}$, $S_{EE}^{4}$), is the non-trivial and positive solution to the following system

(3.10) $$\frac{a^{i}(S_{0}^{i}-S_{EE}^{i})}{{ℛ}_{0}^{i}S_{EE}^{i}}=\frac{a^{j}(S_{0}^{j}-S_{EE}^{j})}{{ℛ}_{0}^{j}S_{EE}^{j}},\;\;1\leq i\neq j\leq 4,\;\sum_{\kappa =1}^{4}S_{EE}^{\kappa }(1-{ℛ}_{0}^{\kappa }-d^{\kappa })+d^{\kappa }S_{0}^{\kappa }=0,$$

where the coefficients

$$a^{\kappa }=\frac{{\bar{\tau }}_{I}^{\kappa }\mu^{\kappa }}{1-{𝒫}_{\theta }^{\kappa }},\;\;d^{\kappa }=\frac{({\bar{\tau }}_{I}^{\kappa }+{\bar{\tau }}_{R}^{\kappa })\mu^{\kappa }}{1-{𝒫}_{\theta }^{\kappa }},\;\;\kappa =1,\cdots ,4,$$

the coefficient ${ℛ}_{0}^{\kappa }$ is defined in Eq (3.2), and $S_{0}^{\kappa }$ is the DFE.

By using a combination of probabilities rather than rates, we interpret the obtained endemic steady states: At the endemic steady state, the migration-in rate, $\mu^{\kappa }(S_{0}^{\kappa }-S_{EE}^{\kappa })$, needs to balance out the migration-out rate, which is a product of the total infected population, $I_{EE}^{\kappa }$, and averaged death rate, ($\left(1-{𝒫}_{\theta }^{\kappa }\right)∕({\bar{\tau }}_{I}^{\kappa }+{\bar{\tau }}_{M}^{\kappa }+{\bar{\tau }}_{R}^{\kappa })$). Thus, at the steady state, we obtain the balanced equation

$$\mu^{\kappa }(S_{0}^{\kappa }-S_{EE}^{\kappa })=\frac{1-{𝒫}_{\theta }^{\kappa }}{{\bar{\tau }}_{I}^{\kappa }+{\bar{\tau }}_{M}^{\kappa }+{\bar{\tau }}_{R}^{\kappa }}I_{EE}^{\kappa },$$

and the total balanced infected population (people who has been infected) is

$$I_{EE}^{\kappa }=\frac{\mu^{\kappa }(S_{0}^{\kappa }-S_{EE}^{\kappa })}{1-{𝒫}_{\theta }^{\kappa }}({\bar{\tau }}_{I}^{\kappa }+{\bar{\tau }}_{M}^{\kappa }+{\bar{\tau }}_{R}^{\kappa })\;.$$

This population $I_{EE}^{\kappa }$ is then distributed to different infected compartments, proportional to the expected infection time span spent (${\bar{\tau }}_{\Delta }^{\kappa }$) in that compartment as defined in Eq (3.5).

## 4. Numerical examples

### 4.1. Baseline model calibration & simulation

The disease transmission rates, $c\beta^{\kappa }$, are difficult to quantify directly and need to be estimated based on more observable quantities. We calibrate the model using the incidence information in Table 2, which describes the situation in Siaya County, Kenya.

The migration rate, $\mu^{\kappa }$ (per capita per day), includes both the baseline (non-iNTS related) death rate and the aging rate, and we assume there is no significant spatial migration in/out of the studied region in this age range [41]. For example, for the children under age 5 (κ=4), $\mu^{4}=\left(10.4∕1000+1∕5\right)∕365\approx 5.8\times 10^{-4}$.

To estimate the transmission rate, $c\beta^{\kappa }$, we match the incidences at the endemic state. To this end, we setup a nonlinear system,

$${\left(\lambda^{\kappa }\right)}_{EE}=c\beta^{\kappa }\frac{\sum_{\kappa =1}^{4}{\left(I_{1}^{\kappa }\right)}_{EE}+{\left(I_{2}^{\kappa }\right)}_{EE}+{\left(I_{HT}^{\kappa }\right)}_{EE}+{\left(A^{\kappa }\right)}_{EE}}{\sum_{\kappa =1}^{4}S_{EE}^{\kappa }+{\left(I_{1}^{\kappa }\right)}_{EE}+{\left(I_{2}^{\kappa }\right)}_{EE}+{\left(I_{HT}^{\kappa }\right)}_{EE}+{\left(A^{\kappa }\right)}_{EE}+{\left(R^{\kappa }\right)}_{EE}}\;\;=c\beta^{\kappa }\frac{\sum_{\kappa =1}^{4}a^{\kappa }(S_{0}-S_{EE}^{\kappa })}{\sum_{\kappa =1}^{4}S_{EE}^{\kappa }+d^{\kappa }(S_{0}-S_{EE}^{\kappa })}=\frac{\text{incidence for group}\;\kappa }{365\times 100,000},\;\kappa =1,\cdots ,4,$$

and the four equations from the system Eq (3.10), and solve for the eight unknowns: the transmission rates $c\beta^{\kappa }$ (product as one variable) and endemic states for susceptible $S_{EE}^{\kappa }$, κ=1,⋯,4.

In 2002–2003, an emerging iNTS epidemic took about three years to peak in a naive West African population [34,42]. Although the reported estimates for the iNTS disease burden are known to be gross underestimates of the actual incidence, they probably capture the underlying trend of the epidemic. We assumed that approximately 20% of the cases were reported. We considered other underreporting factors to verify that the qualitative aspects of predictions were fairly insensitive to this assumption.

We initialized the system of equations by infecting one person and letting the infection spread until 0.1% of the population was infected. We then used this balanced distribution of infected and recovered populations for the t=0 initial conditions for the model. This balanced initial condition gives a naturally distributed infection across different compartments as if the epidemic had emerged from a single infection. The initialization also avoids nonphysical oscillations that can occur when the population is not realistically distributed among the different compartments in the initial conditions.

**Figure 3.**
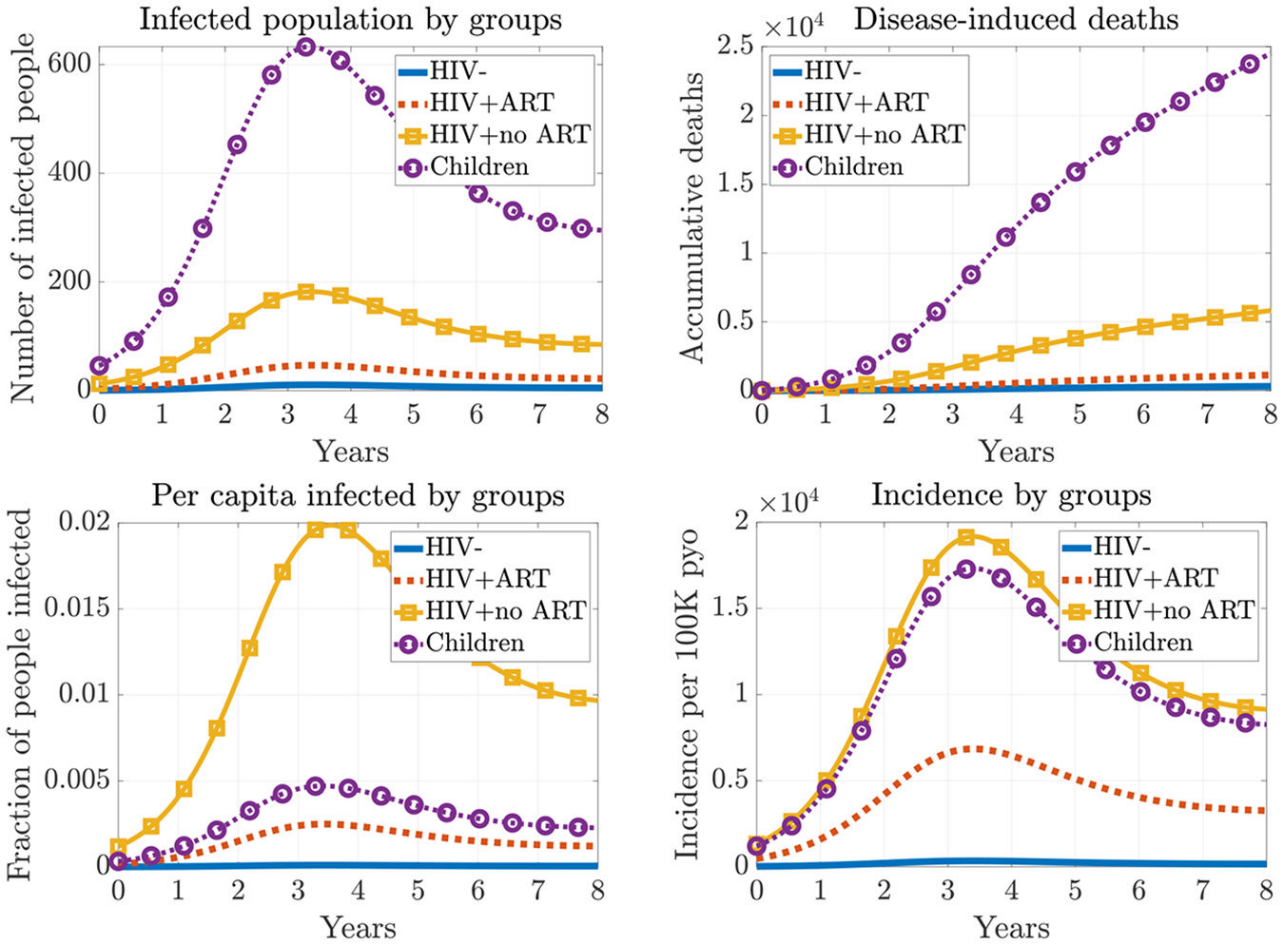
Baseline simulations with 0.1% initial infection in the population. The child group has the most infected cases (top left) and most deaths (top right), and the HIV+ without ART adult group has the highest per capita infection counts (bottom left) and comparable incidence rates with the child group (bottom right).

To study the population cohort that are most impacted by the pathogen, we simulate the infection and death-count curves for different groups in one epidemic. At the baseline scenario, the child group (κ=4) suffers the most from the epidemic as it has the most infection cases throughout the course of the epidemic (Figure 3 top left) and the most accumulative deaths (top right) among all the population cohorts.

On the other hand, the HIV adult without ART group (κ=3) has the highest per capita infection rate (bottom left), which is about four times higher than the rate in children. The incidence rates are comparable between the HIV non-ART adults and child groups (bottom right), which suggests that HIV no-ART adults have a longer infection course than the children have. In fact, we can estimate the average lengths of infection periods, ${\bar{\tau }}_{I}^{\kappa }+{\bar{\tau }}_{M}^{\kappa }$ (defined in Eqs (3.5) and (3.7)), for HIV no-ART adults and children, which are about 26 days and 10 days, respectively. The major difference is due to the substantial period that HIV no-ART adults spend in the asymptomatic stage, which is about 15 days on average.

**Figure 4.**
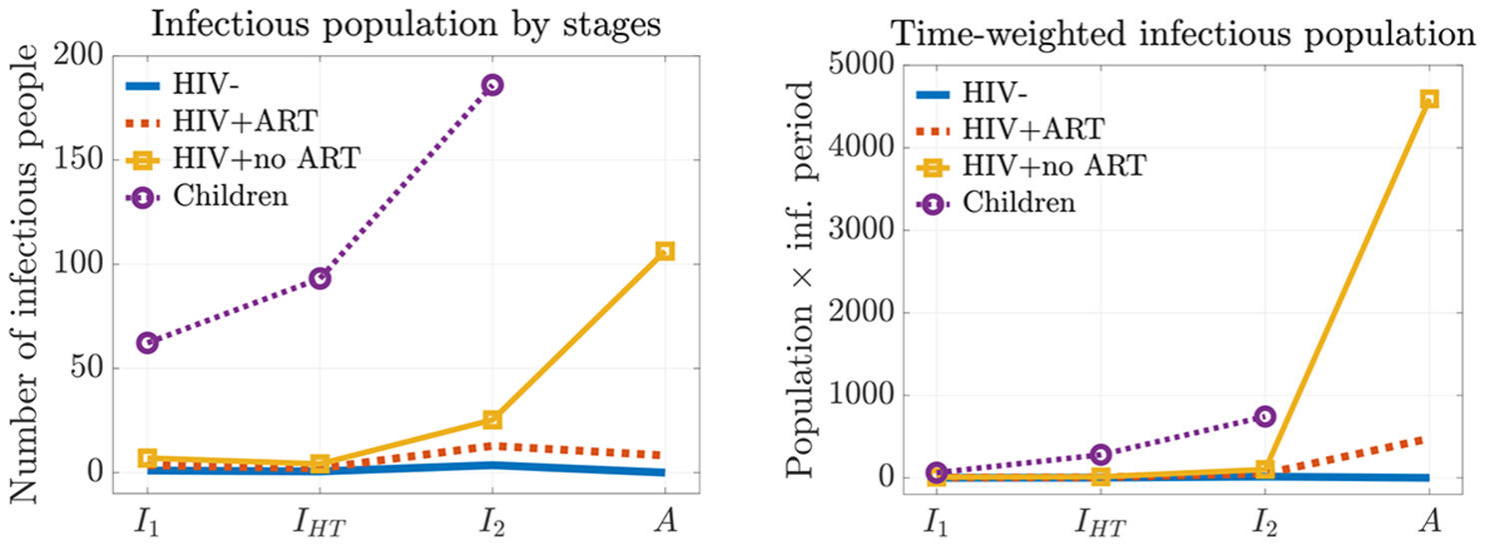
Infectious population by stages near the peak of the epidemic. The $I_{2}$-stage children are the largest infectious population by infection stages (left). However, the asymptomatic HIV+ adults without ART are the largest source of infection, creating most cases in time (right). This adult cohort serves as the driving force of the epidemic that leads to the worst outcome in children. This trend remains the same throughout the epidemic.

We then study the pathogen shedding at different stages to understand the major source of infection. Among all the population cohorts, the child group has the most infectious counts in all its stages (Figure 4 left). However, when we also consider the infectious time spent at different stages, then the asymptomatic HIV+ adults without ART gives the most time-weighted infectious counts (Figure 4 right).

The critical role that HIV+ adults without ART play can also be seen in the basic reproduction numbers for different subgroups (Table 3). We see that among all the population cohorts (column 2 of Table 3), HIV+ no ART adults contribute the most in terms of ${ℛ}_{0}^{\kappa }$. Furthermore, if breaking this contribution to different infectious stages (row 4 of Table 3), the asymptomatic stage of the HIV+ no ART adult group (column 6) has the largest value (6.31). This indicates that these asymptomatic untreated HIV adults serve as the main driving force of the iNTS epidemic.

### 4.2. Sensitivity analysis and mitigation strategies

We are interested in understanding how the risk factors could affect the epidemic course and exploring potential mitigation strategies for iNTS disease control. The sensitivity analysis identifies impactful model parameters and quantifies the relative changes in the quantities of interest (QOIs) with respect to the perturbation on the model parameters of interest (POIs) [43]. We define the normalized sensitivity index of a QOI, q(p), with respect to a POI, p, as

$${𝒮}_{p}^{q}=\frac{p}{q}\times \frac{\partial q}{\partial p}.$$

This index measures a percentage response: if the parameter p changes by x%, then the quantity q changes by ${𝒮}_{p}^{q}\times x\%$. The sign of the index indicates if the response is positively or negatively correlated.

We first consider the QOIs that are related to the source of the infection: the ${ℛ}_{0}$ and the population size of asymptomatic non-ART adults, as suggested by the baseline simulations in Section 4.1. The first two columns of the sensitivity indices in Table 4 show that the coverage of ART for the HIV+ adults ($\sigma_{ART}$) is the most sensitive risk factor: a 1% increase in the ART coverage would reduce the ${ℛ}_{0}$ by 0.56% (from ${ℛ}_{0}=1.08\to 1.074$), and the total number of asymptomatic HIV without ART would be reduced by 7.59% (8 fewer people). This suggests that it would be the most effective to control the source of the infection by improving the coverage of the ART among the HIV-infected adults.

The changes in ${ℛ}_{0}$ (0.56%) and asymptomatic HIV+ no-ART ($A^{3}$ group) adults (7.59%) may look like a small perturbation of the baseline scenario. However, each of these $A^{3}$ adults may create a super spreading event: the $A^{3}$ group consists around 12% of the infected population and are responsible for 74% of the infection (infection cases weighted by the ${ℛ}_{0}$’s in Table 3). Thus, it is critical to reduce this highly infectious group and control the silent shedding.

Among the QOIs related to the disease dynamics and outcomes (columns 3–5 in Table 4), the prevalence of malnutrition in children ($\sigma_{MN}$) is the most sensitive factor: by lowering 1% of the malnutrition prevalence, the total infected population at the peak would be reduced by 10.86% (81 fewer cases, including 68 children), and the accumulative disease-induced deaths through one outbreak season (the endemic state reached around year 8) could be reduced by 7.67% (2450, including 488 adults and 1962 children). Moreover, $\sigma_{MN}$ is the second most sensitive parameter for the other two QOIs (columns 1–2). This suggests that it would be the most effective to reduce malnutrition prevalence in children, who are the largest high-risk population in our baseline simulation, in order to both control the additional shedding to the community and limit human mortality from the epidemic.

Moreover, the peaking time of the epidemic (column 3 in Table 4) has highly sensitive and nonlinear responses to the changes in all the model parameters (see figure below the table). The large magnitudes of the indices suggest that even a small reduction of the risk factors can delay the start of an epidemic, which can be considered as potential strategies to relieve pressure on the healthcare system. The nonlinearity in the response curves indicates that the peaking time of an epidemic will be hard to predict.

## 5. Conclusions

We create and analyze a stage-progression compartmental model to investigate the iNTS epidemic emerging in sub-Saharan Africa. Our model considers multiple age groups in a population with different levels of immune-competence that are associated with the complex risk factors circulating in the region, including the HIV infection, availability of antiretroviral therapy, and high prevalence of malaria infection and malnutrition in children under age 5.

We defined the progression between the infection stages as a function of the branching probabilities at each decision stage and the average time spent within the stage. This approach allows a more straightforward parameter estimation based on the epidemiology literature than directly defining the progression rates. It also gives a more intuitive interpretation during the model analysis.

We derived the basic reproduction number ${ℛ}_{0}$ as a weighted average of the contribution from each population cohort (${ℛ}_{0}^{\kappa }$). Within each population, the contributing ${ℛ}_{0}^{\kappa }$ is the summation of the number of secondary cases generated at each infectious stage weighted by the expected probability of entering the infectious stage. In particular, since asymptomatic adults can have recurrent infections, they create an infinite chain of infection with diminishing probability.

Both the basic reproduction number and the numerical simulations suggest that the HIV+ adults with no ART are the driving force of infection for the epidemic. At the peaking time, they form 12% of the infected population and are responsible for 74% of the infection. Thus, it would be critical and most effective to design the intervention program that targets this particular cohort to control the chain of the infection. On the other hand, our simulations show that largest disease burden is among the children group in terms of the highest infection and death counts. This suggests that reducing the susceptibility among this cohort would reduce the disease burden the most.

The sensitivity analysis identifies that the coverage of ART for the HIV-infected adults, $\sigma_{ART}$, and the malnutrition prevalence in children, $\sigma_{MN}$, are the two most important risk factors that may inform effective disease mitigation. To control the source of the infection, it would the most impactful to improve ART coverage among the HIV+ adults, which would reduce the asymptomatic cohort that drives the epidemic. Children are the largest high-risk population, and it would be most productive to reduce the overall disease burden by lowering the malnutrition prevalence in this cohort. This would improve their immune competence and thus make them less susceptible to the pathogen. To better inform the public health efforts, it will be useful to combine our model with a cost-benefit analysis to optimize mitigation strategies.

This model offers important insights into iNTS dynamics and control. We recognize that our conclusions are based on assumptions, biases, and uncertainties in our model and parameters. Many of these limitations are related to the choice of model parameters. For example, we have assumed that all the parameters are constant, and there is no seasonal variation. In reality, malaria prevalence among children is correlated with the seasonal peak of the rainy season, and the local temperature is also playing a vital role in the mosquito-borne disease. Moreover, the malnutrition prevalence also increases during the rainy season when the household food supplies decrease, and the new season’s crops are growing.

Moreover, by having constant parameters rather than distributions, we didn’t consider the potential super spreading events that may cause small outbreaks in a local area. We have made the assumption that these events are uniformly distributed in time and the mean-field approximation can capture their average impact over the time scales we are considering. It would be worthwhile to validate this assumption in the future by comparing our simple model with a more complex simulation.

Our results will guide us in developing a more detailed individual agent-based model. The agent-based model will account for each individual’s age, treatment history, family unit, and local community spatial mixing. The sensitivity analysis has identified the importance of including comorbidities, such as HIV infection and malnutrition, in our simulation. We will also investigate the impact of different mitigation strategies, seasonality, and local superspreading events.

Thus, before using any model to guide policy, the model parameters need to be carefully reviewed for the local setting. Also, the uncertainty of model predictions must be quantified with respect to the model assumptions. The current model is a preliminary study to investigate the iNTS epidemic, yet it provides a robust framework that could be further extended to incorporate more practical scenarios. We hope that models similar to the one presented here can help inform public health workers to mitigate the disease burden.

## Figures and Tables

**Table 1.** State variables for compartments.

Compartment	Description
$S^{\kappa }$	Susceptible
$I_{1}^{\kappa }$	Infected people with mild symptoms
$I_{HT}^{\kappa }$	Infected people who do home treatment
$I_{2}^{\kappa }$	Infected people with severe symptoms
$I_{MT}^{\kappa }$	Infected people who are admitted to medical facilities
$R^{\kappa }$	People recover from the infection (with temporary immunity)
$A^{\kappa }$	People with asymptomatic carriage of the infection (go back home)
$D^{\kappa }$	People die from the infection
Group index κ	
κ=1	HIV− adults, 25–40 years old
κ=2	HIV+ adults, on ART, 25–40 years old
κ=3	HIV+ adults, not on ART, 25–40 years old
κ=4	Children, ⩽ 5 years old

**Table 2.** Estimated event probabilities and parameters for the branching process. Although we have extensively reviewed the literature and chose parameters that best describe the local population cohorts at hand, we could not identify solid references for the parameters referenced by $EO^{*}$ (expert opinion). These parameter values are based on extensive discussions with experts and their studies [36-39] in University of New Mexico-Kenya Programs in Siaya, Kenya, Africa.

	Description	HIV− Adults (κ=1)	HIV+ ART (κ=2)	HIV+ no ART (κ=3)	Children (κ=4)	Reference
$c\beta^{\kappa }$	Transmission rate	4.6 × 10^−3^	0.092	0.257	0.232	Calibrated
$\mu^{\kappa }$	Migration rate (mortality+aging) per day	2.0 × 10^−4^	3.7 × 10^−4^	9.2 × 10^−4^	5.8 × 10^−4^	Derived
	- Mortality rate per 1000 people per year	5.02	70	270	10.4	[25, 26]
	- Aging rate per year	1/15	1/15	1/15	1/5	Derived
$S_{0}^{\kappa }$	Population size (Siaya County)	106202	19171	10784	141752	[27]
-	Incidence, per 100,000 population per year	37	739	2070	1870	[28, 29]
$\sigma_{MA}$	Prevalence of malaria epidemic in children	-	-	-	2%	[30]
$\sigma_{MN}$	Prevalence of malnutrition in children	-	-	-	40%	[26]
$\sigma_{HIV}$	Prevalence of HIV epidemic	$\sigma_{HIV}^{A}=22\%$	$\sigma_{HIV}^{A}=22\%$	$\sigma_{HIV}^{A}=22\%$	$\sigma_{HIV}^{C}=2\%$	$EO^{*}$
$\sigma_{ART}$	Coverage of ART for HIV adults		64%		-	$EO^{*}$
$\sigma_{MAT}$	Maternal trans. rate for non-ART adults	-	-	30%	-	[31]
Without the effect of migration, the raw estimates for disease parameters…						
$P_{1H}^{0}$	Fraction of $I_{1}$ do self-treatment	20%	20%	20%	50%	$EO^{*}$
$P_{12}^{0}$	Fraction of $I_{1}$ do not do self-treatment	80%	80%	80%	50%	$EO^{*}$
$P_{HR}^{0}$	Fraction of $I_{HT}$ recover	50%	40%	10%	50%	$EO^{*}$
$P_{HA}^{0}$	Fraction of $I_{HT}$ become asymp.	-	4% [32]	35%*	-	$EO^{*}$
$P_{H2}^{0}$	Fraction of $I_{HT}$ become $I_{2}$	50%	56%	55%	50%	$EO^{*}$
$P_{2M}^{0}$	Fraction of $I_{2}$ do medical-treatment	90%	90%	90%	90%	$EO^{*}$
$P_{2D}^{0}$	Fraction of $I_{2}$ do not get medical-treatment	10%	10%	10%	10%	$EO^{*}$
$P_{MD}^{0}$	Fraction of $I_{MT}$ die	11% [33]	11% [33]	47% [6]	22% [34]	$EO^{*}$
$P_{MA}^{0}$	Fraction of $I_{MT}$ become asymp.	-	4%	35%*	-	$EO^{*}$
$P_{MR}^{0}$	Fraction of $I_{MT}$ recover	89%	85%	18%	78%	$EO^{*}$
$\tau_{1}^{0}$	Time spent in $I_{1}$ (infected → lightly sick)	1 d	1 d	1 d	1 d	$EO^{*}$
$\tau_{H}^{0}$	Time spent in self-treatment	3 d	3 d	3 d	3 d	$EO^{*}$
$\tau_{2}^{0}$	Time spent in $I_{2}$ (lightly sick → heavily sick)	4 d	4 d	4 d	4 d	$EO^{*}$
$\tau_{M}^{0}$	Time spent in medical-treatment	7 d	7 d	7 d	7 d	$EO^{*}$
$\tau_{R}^{0}$	Time of immunity period after recovery	1 yr	1 yr	1 yr	2 m	$EO^{*}$
$\tau_{A}^{0}$	Time of being asymptomatic	-	59 d [32]	45 d [32] (60 d [35])	-	$EO^{*}$
The adjusted parameter values for migration…						
$\tau_{*}$	Time spent in stage *	${\left(1∕\tau_{*}^{0})+\mu^{\kappa }\right)}^{-1}$				Derived
$P_{*N}$	Fraction of migration at stage *	$\mu^{\kappa }\times \tau_{*}$				Derived
$P_{ij}$	Fraction of people at stage i move to stage j	$P_{ij}^{0}*(1-P_{*N})$				Derived

**Table 3.** At the baseline scenario, the basic reproduction number for the entire population is ${ℛ}_{0}=1.08$, which is a weighted average of the basic reproduction number from each cohort, ${ℛ}_{0}^{\kappa }$ (column 2). For each cohort, ${ℛ}_{0}^{\kappa }$ is the summation of the contribution from all the infectious stages (columns 3–6). The most infectious group is the asymptomatic HIV+ no ART adults.

	${ℛ}_{0}^{\kappa }$	$I_{1}$	$I_{HT}$	$I_{2}$	A
HIV− adults (κ=1)	0.03	5 × 10^−3^	3 × 10^−3^	0.02	-
HIV+ART adults (κ=2)	0.77	0.10	0.06	0.37	0.24
HIV+ no ART adults (κ=3)	**8.47**	0.41	0.25	1.50	**6.31**
Children (κ=4)	1.35	0.25	0.37	0.74	-

**Table 4.** Local (normalized) sensitivity indices, ${𝒮}_{p}^{q}$, for QOIs (top labels) with respect to POIs (left column). For each QOI, the index for the most sensitive POI is in bold. For all the QOIs considered, the prevalence of malnutrition, $\sigma_{MN}$, and coverage of ART for HIV+ adults, $\sigma_{ART}$, are the most sensitive parameters. The peaking time gives a nonlinear response to the perturbation in model parameters (right plot) with the largest indices. Hence, it is the most unpredictable quantity.

Normalized sensitivity indices						
_POI_╲^QOI^	${ℛ}_{0}$	Asymp. no-ART adults (peak)	Peaking time	Total infect. at peak	Total infect. at endemic	Accum. deaths
$\sigma_{MN}$	0.53	5.84	−**5.94**	**10.86**	**7.12**	**7.67**
$\sigma_{ART}$	−**0.56**	−**7.59**	5.01	−9.98	−6.70	−7.25
$\sigma_{HIV}$	0.39	5.21	−3.54	7.18	4.88	5.20
$\sigma_{MA}$	0.07	0.82	−1.24	1.52	1.00	1.07
$\sigma_{MAT}$	0.04	0.43	−0.42	0.81	0.53	0.57
Trend	Linear	Linear	Nonlinear	Linear	Linear	Linear